# 5-ALA in Suspected Low-Grade Gliomas: Current Role, Limitations, and New Approaches

**DOI:** 10.3389/fonc.2021.699301

**Published:** 2021-07-30

**Authors:** Barbara Kiesel, Julia Freund, David Reichert, Lisa Wadiura, Mikael T. Erkkilae, Adelheid Woehrer, Shawn Hervey-Jumper, Mitchel S. Berger, Georg Widhalm

**Affiliations:** ^1^Department of Neurosurgery, Medical University of Vienna, Vienna, Austria; ^2^Center for Medical Physics and Biomedical Engineering, Medical University of Vienna, Vienna, Austria; ^3^Christian Doppler Laboratory OPTRAMED, Medical University of Vienna, Vienna, Austria; ^4^Department of Neurology, Institute for Neuropathology and Neurochemistry, Medical University of Vienna, Vienna, Austria; ^5^Department of Neurological Surgery, University of California San Francisco (UCSF), San Francisco, CA, United States

**Keywords:** 5-ALA, suspected LGG, anaplastic foci, spectroscopic PpIX analysis, fluorescence lifetime imaging, confocal microscopy

## Abstract

Radiologically suspected low-grade gliomas (LGG) represent a special challenge for the neurosurgeon during surgery due to their histopathological heterogeneity and indefinite tumor margin. Therefore, new techniques are required to overcome these current surgical drawbacks. Intraoperative visualization of brain tumors with assistance of 5-aminolevulinic acid (5-ALA) induced protoporphyrin IX (PpIX) fluorescence is one of the major advancements in the neurosurgical field in the last decades. Initially, this technique was exclusively applied for fluorescence-guided surgery of high-grade glioma (HGG). In the last years, the use of 5-ALA was also extended to other indications such as radiologically suspected LGG. Here, we discuss the current role of 5-ALA for intraoperative visualization of focal malignant transformation within suspected LGG. Furthermore, we discuss the current limitations of the 5-ALA technology in pure LGG which usually cannot be visualized by visible fluorescence. Finally, we introduce new approaches based on fluorescence technology for improved detection of pure LGG tissue such as spectroscopic PpIX quantification fluorescence lifetime imaging of PpIX and confocal microscopy to optimize surgery.

## Introduction

Neurosurgical resection constitutes the primary treatment in low-grade gliomas (LGG), but still remains challenging due to their histopathological heterogeneity as well as infiltrative growth pattern into the surrounding brain parenchyma (1–5). Thus, incomplete resection of LGG and histopathological undergrading of gliomas is not uncommon in the routine neurosurgical practice (6–14). In the last decades, different techniques were introduced into the neurosurgical operating room for improved intraoperative visualization of LGG tissue such as neuronavigation with multimodal imaging data, intraoperative MRI and advanced ultrasound (15–24).

Aside from these techniques, intraoperative visualization of brain tumor tissue with assistance of 5-aminolevulinic acid (5-ALA) induced fluorescence represents one of the most powerful methods for visualization of tumor tissue during surgery (7, 8, 25–31). Initially, this innovative fluorescence technique was exclusively applied during tumor resection of high-grade gliomas (HGG) (7, 8, 25, 29, 32). According to the data from a randomized controlled multicenter phase III trial, the rate of complete resections as well as the progression-free survival was significantly higher in HGG patients with 5-ALA fluorescence-guided surgery as compared to conventional white-light microscopy (7). Therefore, the 5-ALA fluorescence technique is current standard for resection of HGG in many neurosurgical departments around the world (7). Based on these promising observations in HGG, 5-ALA was increasingly applied also in patients with radiologically suspected LGG in the last years (24, 33–36). Here, we discuss the current role, limitations and new approaches of 5-ALA fluorescence during surgery of suspected LGG.

## Low-Grade Gliomas

LGG represent a heterogenous group of astrocytic and oligodendroglial tumors and account for approximately 20% of all primary brain tumors with an incidence rate of 5.2 per 100 000 persons per year (5, 37–41). These tumors predominately affect a younger patient population in the 2^nd^ to 4^th^ decade (5). The median survival rate of patients suffering from LGG ranges from 5 to 13 years (5, 39–42). This wide range of survival rates in LGG are most likely due to differences in clinical, histopathological and molecular/genetic factors. In this sense, patient age and clinical performance status as well as histopathological and molecular genetic factors such as 1p19q co-deletion, isocitrate dehydrogenase (IDH) mutational status, O6-methylguanine-methyltransferase (MGMT) promotor methylation status and alpha thalassemia/intellectual disability X-linked (ATRX) mutation play an important role for patient prognosis in such tumors (13, 40, 43, 44). Additionally, several studies demonstrated the importance of the extent of resection (EOR) on progression-free and overall survival in patients suffering from LGG (5, 11, 21, 45–48). Thus, the neurosurgical aim of surgery in LGG represents maximal safe resection with preservation of neurological function to allow optimal patient prognosis (11, 45–48).

Magnetic resonance imaging (MRI) represents the gold standard for precise diagnosis of suspected LGG (49–57). On T1-weighted sequences, suspected LGG shows an hypointense lesion usually without contrast-media enhancement (49–57). A circumscribed area of contrast-enhancement within an otherwise non-enhancing tumor frequently indicates the occurrence of intratumoral malignant progression within the so-called “anaplastic focus” (6, 10, 58–64). For preoperative identification of intratumoral anaplastic foci within initially suspected LGG, advanced MRI techniques such as MRI spectroscopy, diffusion-weighted imaging, perfusion-weighted imaging as well as positron emission tomography (PET) using amino acids are powerful techniques (6, 23, 24, 54, 56, 59, 65).

## Current Techniques and Limitations of Intraoperative LGG Visualization

At present, the use of intraoperative neuronavigation systems to optimize surgery of suspected LGG is routinely applied (22–24, 66). In this sense, navigation with T2-weighted/FLAIR sequences supports the neurosurgeon to improve intraoperative visualization of LGG tissue especially at the tumor margin in order to achieve the neurosurgical goal of a complete tumor resection according to the current Response Assessment in Neuro-Oncology (RANO) (51, 67, 68).

Although neuronavigation systems are routinely applied, this technique lacks accuracy in the course of glioma resection due to the so-called “brain-shift” leading to significant inaccuracy in image guidance since neuronavigation is based on preoperative image data (69–71). Thus, the occurrence of brain-shift during surgery of suspected LGG might impede precise detection of the tumor margin and the anaplastic focus (24, 58, 71, 72). Furthermore, insufficient intraoperative identification of LGG tissue as well as insufficient differentiation of intratumoral focal HGG tissue representing the anaplastic focus within LGG tissue represents a major challenge for the neurosurgeon (73, 74). Therefore, incomplete resection is reported in up to 88% of cases in surgery of LGG and histopathological undergrading is not uncommon in the routine neurosurgical practice (11, 13, 48, 75).

To overcome these current limitations of glioma surgery, intraoperative MRI was introduced into the neurosurgical field for improved visualization of residual tumor tissue during resection of LGG (16–18, 62, 76–79). This powerful intraoperative technique demonstrated to significantly increase the rate of complete glioma resections (80). However, this technique suffers from specific limitations as intraoperative MRI is not widely available due to its high costs (81). Moreover, specific departments routinely apply intraoperative ultrasound for real-time visualization of tumor tissue during LGG surgery (20, 82). Nevertheless, this technique is dependent from large experience of the performing neurosurgeon and thus this tool is infrequently used (82, 83).

Consequently, new and innovative widely available intraoperative techniques are required to overcome the above-mentioned current limitations in order to improve visualization of LGG tissue and intratumoral heterogeneity of diffusely infiltrating gliomas during surgery.

## Fluorescence-Guided Surgeryin Neurosurgery

Intraoperative visualization of brain tumor tissue with the assistance of fluorescence is unaffected by brain-shift and thus might overcome the current limitations of surgery in suspected LGG (7, 32, 84, 85). Already in 1948, Moore et al. reported their first observations of visible fluorescence using fluorescein in different types of brain tumors (86). Since 1992, the fluorescent dye 5-ALA was increasingly applied for intraoperative visualization of tumor tissue in different medical disciplines such as urology and dermatology (87, 88). In 1998, Stummer et al. reported the use of this well-tolerated fluorescent dye for fluorescence-guided surgery in the first patients in the neurosurgical field as well (89). In 2000, the authors published the first clinical study including 52 consecutive patients suffering from HGG (8). In this study, Stummer et al. observed strong 5-ALA fluorescence in HGG tissue, whereas normal brain tissue did not show any visible fluorescence during surgery (8). Based on these first promising findings, Stummer et al. initiated in a further step a randomized controlled multicenter phase III trial investigating the impact of 5-ALA fluorescence-guided resection on EOR and progression-free survival in HGG (7). According to their data, this study showed that 5-ALA fluorescence-guided surgery improves the rate of complete resection of the contrast-media enhancing tumor resulting in a significantly prolonged progression-free survival as compared to conventional white-light resections (7). Consequently, 5-ALA was approved for resection of HGG in the European Union in 2007. Following this approval, fluorescence-guided surgery using 5-ALA became more and more attractive in the neurosurgical field as it is relatively inexpensive, widely available and has little side effects (7, 29, 30, 32). Ten years later, 5-ALA was approved by the FDA for intraoperative visualization of suspected HGG in the United States as well (85).

## Methods

In this review, we performed a literature search to discuss the current role, limitations, and new approaches of 5-ALA fluorescence during surgery of suspected LGG. For this purpose, we performed a literature research using Pubmed to screen the MEDLINE database for relevant publications. To cover all relevant publications, we used different search criteria such as “5-ALA”, “glioma”, “LGG” and “PET”. Additionally, we also included “HGG” in our search criteria as we aimed to identify cohorts including “radiologically suspected LGG” which may present as neuropathologically confirmed HGG. Therefore, our literature review also covers cohorts including HGG as well as LGG cases with the application of 5-ALA.

## 5-ALA Fluorescence in LGG

Based on these auspicious findings in HGG, the application of 5-ALA was also investigated in LGG in the last years. In this sense, Ishihara et al. reported in 2007 altogether 6 patients including 2 LGG, 2 HGG and 2 GBM that were investigated *ex-vivo* after 5-ALA administration (90). Interestingly, only samples from HGG and GBM demonstrated macroscopic visible 5-ALA fluorescence, whereas in all LGG samples no visible fluorescence was observed (90). Additionally, Stummer et al. described one single patient with focal malignant transformation showing visible 5-ALA fluorescence in the contrast-enhancing area, but no fluorescence in the non-enhancing LGG part (89). As these studies only included single cases, larger patient cohorts were needed.

In 2010, Widhalm et al. evaluated in the first clinical study the intraoperative application of 5-ALA in 19 cases of radiologically suspected LGG (58). In this first *in-vivo* study, the 5-ALA fluorescence status was analyzed during resection and demonstrated that a subgroup of these suspected LGG showed visible fluorescence in a circumscribed intratumoral area (58). Interestingly, histopathological analysis revealed 8 LGG cases which did not show any visible 5-ALA fluorescence (58). Notably, all tumors showing visible intratumoral fluorescence were histopathologically classified as WHO grade III gliomas resulting in a positive predictive value of focal 5-ALA fluorescence for WHO grade III glioma of 100% (58). Additionally, gliomas with visible 5-ALA fluorescence showed a significantly higher proliferation rate (MIB-1: 20 *vs* 6%, p=0.001) compared to gliomas without visible fluorescence (58). In conclusion, Widhalm et al. suggested 5-ALA fluorescence as a promising tool to intraoperatively visualize anaplastic foci within initially suspected LGG (58). In addition, the authors hypothesized that 5-ALA fluorescence might optimize tissue sampling and subsequently improves postoperative treatment allocation in suspected LGG patients (58).

In a further study conducted by Ewelt et al. these initial findings suggesting that visible 5-ALA fluorescence in suspected LGG is a predictive marker for high-grade histology were confirmed in an independent cohort (91). Of these 30 included tumors, 13 were diagnosed as WHO grade II, 15 as WHO grade III and two as WHO grade IV gliomas (91). Visible fluorescence was observed in 13 of 30 patients in this study (91). According to histopathological analysis, the majority of WHO grade III and IV gliomas demonstrated visible fluorescence (2 of 2 GBM, 10 of 15 WHO grade III gliomas; 70%) (91). In contrast, only one of 11 WHO grade II gliomas showed visible fluorescence (91).

In a subsequent clinical study, Widhalm et al. analyzed 59 patients with a suspected LGG consisting of 26 HGG and 33 LGG (26). In this study, 85% of all WHO grade III gliomas showed focal fluorescence, whereas 91% of WHO grade II gliomas revealed no visible fluorescence (26). According to detailed histopathological analysis, the authors found a significantly higher mitotic rate, cell density, nuclear pleomorphism and proliferation rate in intratumoral areas with focal 5-ALA fluorescence compared to areas without visible fluorescence (26). These findings resulted in a positive predictive value of visible 5-ALA fluorescence for WHO grade III histology of 85% (26). In this study, the authors confirmed their initial findings of their pilot study that 5-ALA is a powerful and reliable intraoperative marker for anaplastic foci identification independent of brain-shift (26). Additionally, this study reported a significant correlation of focal 5-ALA fluorescence with specific histopathological parameters of anaplasia (26).

The largest patient cohort up to date of 166 tumors lacking characteristic GBM imaging features included 82 WHO grade II, 76 WHO grade III and 8 WHO grade IV gliomas (92). This study was published in 2016 by Jaber et al. and correlated 5-ALA with MRI, PET, proliferation index and molecular genetics (92). According to the data, WHO grade histology and proliferation index correlated with visible 5-ALA fluorescence, however, no correlation was found with MGMT promoter methylation status, IDH1 mutational status or 1p19q co-deletion (92). Furthermore, fluorescing WHO grade III gliomas showed a significantly higher proliferation index as compared to tumors without visible fluorescence (92). Interestingly, no difference of proliferation index was noted in WHO grade II gliomas with visible fluorescence and no visible fluorescence, respectively (92). In brief, this study confirmed that visible 5-ALA fluorescence is associated with high-grade histology and increased proliferation index (92). However, the authors concluded that further analysis to identify the impact of 5-ALA fluorescence in a subgroup of WHO grade II gliomas are needed (92).

An additional study published in 2017 by Saito et al. evaluated the association between 5-ALA fluorescence and proliferation rate as well as molecular markers including IDH1 mutational status and 1p19q co-deletion in a series of WHO grade II, III and IV gliomas (93). In this study, univariate analysis indicated that 5-ALA fluorescence is significantly related to proliferation rate as well as IDH1 mutational status and 1p19q co-deletion (93). According to multivariate analysis, only IDH1 status remained statistically significant (93). In detail, gliomas with visible 5-ALA fluorescence showed a significantly higher rate of IDH1 wildtype tumors (93). However, this high rate of visible fluorescence in IDH1 wildtype tumors might be explained by the high number of WHO grade IV gliomas with IDH1 wildtype included in this analysis (93).

In 2019, Jaber et al. evaluated the impact of visible 5-ALA induced fluorescence in a study cohort including only histopathologically confirmed pure LGG (36). In this study, 74 patients with pure LGG were analyzed and visible 5-ALA fluorescence was observed in 16 of 74 (22%) cases (36). Interestingly, progression-free survival, malignant transformation-free survival and overall survival were shorter in tumors showing visible 5-ALA fluorescence as compared to non-fluorescing LGG (36). However, in this study only overall survival was statistically significant (36). According to these findings, the authors proposed that postoperative adjuvant therapies might be early considered in patients with histopathologically confirmed LGG and visible 5-ALA fluorescence (36). Additionally, Jaber et al. suggested shorter intervals during MRI follow-up examinations should be considered (36).

**Figure 1:**
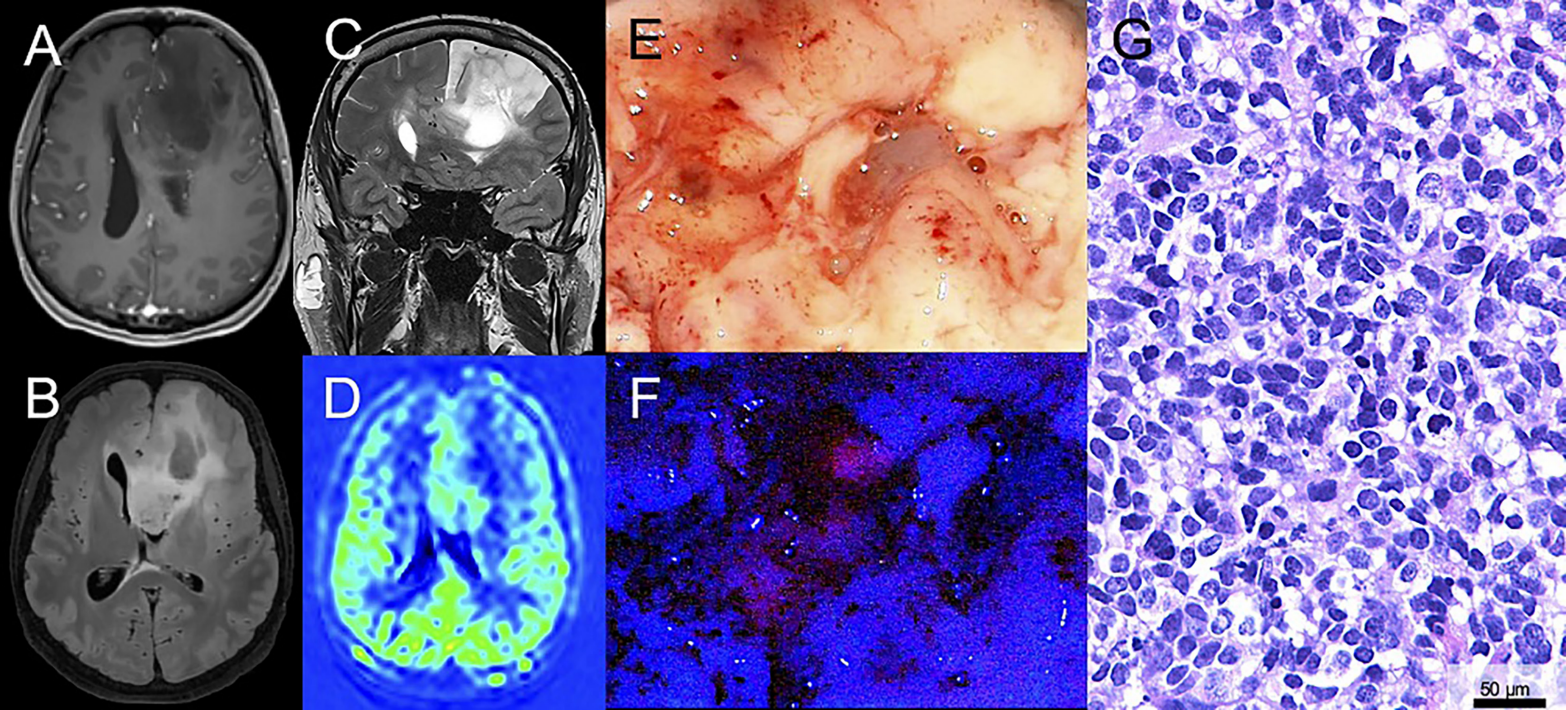
Illustrative case of a patient with a suspected low-grade glioma (LGG) with surgical resection after 5-ALA administration. **(A)** Magnetic resonance imaging of a 43-year-old male patient reveals a suspected LGG in the left frontal lobe. On T1-weighted sequences no significant contrast-media enhancement is observed. **(B, C)** On fluid-attenuated inversion recovery (FLAIR) and T2-weighted sequences the lesion is hyperintense. **(D)** Perfusion imaging shows no hyperperfusion. **(E)** During surgical tumor resection, conventional white-light microscopy was used. **(F)** Additionally, the microscope was repeatedly switched to violet-blue excitation light and an intratumoral area of visible fluorescence was detected. **(G)** Histopathological analysis revealed an anaplastic astrocytoma WHO grade III (IDH mutated).

In a further study in the same year, Goryaynov et al. reported the presence of visible 5-ALA fluorescence in a markedly higher portion of LGG patients (52%) (94). However, this study not only included diffusely infiltrating gliomas WHO grade II, but also pilocytic astrocytoma, gemistocytic astrocytoma and desmoplastic infantile ganglioglioma (94). With regard to a subgroup analysis, only 29% of astrocytoma WHO grade II showed visible fluorescence which is in accordance with earlier publications (26, 36, 58, 94). An overview of the literature is given in **Table 1** and an illustrative case is provided in **Figure 1**.

**Table 1:** Studies in the literature with primary focus on visible 5-ALA fluorescence including LGG (WHO grade II).

Publication	Year	Total	Histopathological diagnosis		Positive 5-ALA Fluorescence	
		*n*	*n*		*n*	*(%)*
						
*Ishihara et al.*	2007	6	2	WHO° II	0/2	(0)
			2	WHO° III	2/2	(100)
			2	WHO° IV	2/2	(100)
						
*Widhalm et al.*	2010	17	8	WHO° II	0/8	(0)
			9	WHO° III	8/9	(89)
						
*Ewelt et al.*	2011	30	13	WHO° II	1/13	(8)
			15	WHO° III	10/15	(67)
			2	WHO° IV	2/2	(100)
						
*Widhalm et al.*	2013	59	33	WHO° II	4/33	(12)
			26	WHO° III	23/26	(88)
						
*Jaber et al.*	2016	166	82	WHO° II	19/82	(23)
			76	WHO° III	66/76	(87)
			8	WHO° IV	7/8	(88)
						
*Saito et al.*	2017	60	8	WHO° II	2/8	(25)
			17	WHO° III	9/17	(53)
			35	WHO° IV	31/35	(89)
						
*Jaber et al.*	2019	74	74	WHO° II	16/74	(22)
						
*Goryaynov et al.*	2019	27	20	WHO° II	7/20	(35)
			4	Pilocytic astrocytoma	4/4	(100)
			2	Gemistocytic astrocytoma	2/2	(100)
			1	Desmoplastic infantile ganglioglioma	1/1	(100)

5-ALA, 5-aminolevulinic acid; WHO, World Health Organization.

## 5-ALA Fluorescence and PET Imaging

In the literature, different studies also correlated PET imaging with visible 5-ALA fluorescence in diffusely infiltrating gliomas (36, 92, 95–98). In this sense, Stockhammer et al. first reported in 2009 that the standardized uptake value of PET related to normal brain using ^18^F-fluoroethyl-L-tyrosine (FET) was significantly higher in tumor areas with 5-ALA fluorescence as compared to normal brain with absence of visible fluorescence (95). This study cohort consisted mainly of patients suffering from glioblastomas (n=11 of 13 patients) (95).

In a study published in 2010 including only WHO grade II and III gliomas, Widhalm et al. correlated PET imaging using ^11^C‐methionine (MET) with focal 5-ALA fluorescence (58). In this study, the maximal tumor to normal brain ratio (PET_max_) was significantly higher in the focal 5-ALA fluorescence glioma group as compared to gliomas with lack of fluorescence (58). Furthermore, the authors observed that focal fluorescence always correlated topographically with the MET-PET hotspot verified by intraoperative navigation (58). In a subsequent study with a larger patient cohort, Widhalm et al. confirmed this interesting observation and reported that focal fluorescence correlated topographically in all gliomas with a distinct PET hotspot (26). These data indicate that focal 5-ALA fluorescence is able to intraoperatively identify intratumoral areas with highest metabolic activity according to MET-PET (26, 58). Therefore, the authors advocate the combined use of PET imaging and 5-ALA fluorescence to optimize tissue sampling during surgery of suspected LGG (26, 58). In this sense, PET is a clinically reliable method for preoperative identification of anaplastic foci within suspected LGG and 5-ALA fluorescence is subsequently useful for intraoperative detection of such PET hotspots independent of brain-shift (26, 58).

In a subsequent study published in 2011 Floeth et al. investigated the preoperative FET-PET, contrast media enhancement on MRI and intraoperative 5-ALA fluorescence in 38 glioma samples (33). The study cohort consisted of biopsies from 17 LGG and 21 HGG (33). In biopsies of HGG, 86% of cases showed a metabolic hotspot on FET-PET as compared to visible 5-ALA fluorescence/contrast-enhancing area on MRI which was present in 57% of cases (33). Interestingly, all gliomas with visible 5-ALA fluorescence showed a metabolic hotspot on FET-PET (33). Despite this positive correlation of visible 5-ALA fluorescence and FET-PET, the authors postulated that FET-PET is more sensitive for glioma tissue detection especially in LGG compared to 5-ALA fluorescence (33).

Another study by Ewelt et al. published in 2011 analyzed the FET-PET uptake and its correlation with 5-ALA fluorescence in 30 gliomas (91). In this study, all tumors with intraoperative visible fluorescence were positive on preoperative FET-PET (91). Moreover, FET-PET/5-ALA positive biopsies were found in 71% of HGG, whereas this constellation was observed in only 8% of LGG (91). Furthermore, the FET-PET tracer uptake was significantly higher in tissue samples with visible fluorescence (FET-PET uptake always >1.6) as compared to samples with absence of fluorescence (91). Therefore, the authors concluded in this study that a positive preoperative FET-PET is highly predictive for visible 5-ALA fluorescence during glioma surgery (91). In a further study, this group reported in 2016 that a FET-PET uptake ratio >1.85 predicted visible 5-ALA fluorescence in gliomas with absence of characteristic glioblastoma features based on preoperative MRI (92).

A detailed histopathological analysis of cell density in 11 glioma patients was performed in 2012 by Arita et al. and the authors correlated cell density to MET-PET and 5-ALA fluorescence (99). In this study, cell density was observed to correlate with MET-PET tracer uptake and positive 5-ALA fluorescence (99). Interestingly, MET-PET uptake showed no correlation with areas with positive or negative fluorescence in this study (99). However, this study was limited by its small sample size of included patients (n=11 patients) (99). An overview of the literature is given in **Table 2**.

**Table 2:** Studies in the literature with comparison of PET and visible 5-ALA fluorescence including LGG (WHO grade II).

Publication	Year	Total	Histopathological diagnosis		Positive 5-ALA fluorescence		PET tracer	PET T/N ratio	
		*n*	*n*		*n*	*(%)*	* *	*5-ALA pos*	*5-ALA neg*
									
*Stockhammer et al.*	2009	13	1	WHO° II	1/1	(100)	FET	2.321	1.142
			1	WHO° III	1/1	(100)		***p<0.0001***	
			11	WHO° IV	11/11	(100)			
									
*Widhalm et al.*	2010	17	8	WHO° II	0/8	(0)	MET	2.3	1.2
			9	WHO° III	8/9	(89)		***p=0.037***	
									
*Floeth et al.*	2011	30	13	WHO° II	1/13	(8)	FET	*n.d.*	*n.d.*
			15	WHO° III	8/15	(53)			
			2	WHO° IV	2/2	(100)			
									
*Ewelt et al.*	2011	30	13	WHO° II	1/13	(8)	FET	2.82	1.33
			15	WHO° III	9/15	(60)		***p=0.026***	
			2	WHO° IV	2/2	(100)			
									
*Arita et al.*	2012	11	2	WHO° II	*n.d.*	*n.d.*	MET	1.66	1.41
			2	WHO° III				*p=0.367*	
			7	WHO° IV					

5-ALA, 5-aminolevulinic acid; FET, O-[2- (18F)fluoroethyl]-L-tyrosine; MET, methionine; n.d., no data; PET, positron emission tomography. T/N ratio, tumor to normal brain ratio; WHO, World Health organization. Bold = significant.

## Current Limitations of 5-ALA in Suspected LGG

Although 5-ALA fluorescence-guided procedures represent one of the most important advancements in the neurosurgical field, specific limitations are associated with this technique. First of all, the vast majority of pure LGG do not produce sufficient 5-ALA induced PpIX accumulation and thus visible fluorescence is usually absent during surgery in these tumors (26, 58, 91, 100). Therefore, 5-ALA is usually not useful for improved definition of the tumor margin during surgery of pure LGG (75, 101, 102). Furthermore, 5-ALA is not capable to visualize a subgroup of suspected LGG with presence of an intratumoral anaplastic focus by visible fluorescence during surgery (58, 91). Finally, visible 5-ALA induced fluorescence underlies a subjective observation based on the performing neurosurgeon and is thus associated with an interobserver variability (103, 104). Thus, intratumoral areas with only vague 5-ALA fluorescence might not be recognized by certain neurosurgeons. Consequently, further advancements of this innovative fluorescence technique are necessary to overcome these current limitations in the future.

## Quantitative Spectroscopic Measurement of 5-ALA Induced PpIX Accumulation

In the last years, novel technologies for improved fluorescence detection in brain tumors were introduced in the neurosurgical field. One of the most promising methods represents quantitative spectroscopic analysis of 5-ALA induced PpIX accumulation. In this sense, spectroscopic analysis is capable to measure the characteristic fluorescence signal of PpIX with typical emission peaks at 635nm and 710nm (90, 102, 104–106).

In a study performed by Utsuki et al. in 2006, the authors analyzed the potential of intraoperative laser spectroscopy in 6 patients with brain tumors (105). This promising approach was able to detect tumor tissue by laser spectroscopy at a peak of 636 nm after 5-ALA administration despite the absence of visible fluorescence (105).

In a further study, Ishihara et al. reported an *ex-vivo* quantitative analysis of 5-ALA induced PpIX fluorescence intensity using spectroscopy in 2007 (90). In this study, 65 samples of 6 glioma patients (2 WHO grade II, 2 WHO grade III and 2 WHO grade IV gliomas, respectively) were quantitatively analyzed and correlated with fluorescence intensity and specific histopathological criteria (90). According to the data, proliferation index, CD31-mircovessel density and the vascular endothelial growth factor correlated with spectroscopic fluorescence intensity (90). These observations indicated that a higher proliferation rate might trigger 5-ALA fluorescence as the proliferation index was the strongest histopathological marker correlated with fluorescence intensity (90). Most importantly, tumor tissue of LGG with absence of visible fluorescence could be detected by quantitative spectroscopic analysis of fluorescence intensity (90).

In 2011, Valdes et al. reported a quantitative *ex-vivo* analysis using “PpIX fluorimetry” to measure 5-ALA induced PpIX concentrations in 23 patients with low-grade and high-grade gliomas (107). Interestingly, none of the four WHO grade I gliomas and only one of two included WHO grade II gliomas showed visible fluorescence (107). In this study, the authors analyzed PpIX concentrations measured by PpIX fluorimetry and the proliferation rate of all together 133 tissue samples (107). According to the data, Valdes et al. found significantly higher levels of PpIX concentrations and proliferation rates in samples with visible fluorescence as compared to non-fluorescing specimens (107). It is of note that 40% of the tumor positive samples with absence of visible fluorescence with conventional 5-ALA microscopy demonstrated significant PpIX concentrations (>0.1 µg/mL) (107). These *ex-vivo* data demonstrated the potential ability of quantitative spectroscopic PpIX analysis to visualize also non-fluorescing tumor tissue as well as anaplastic foci within suspected LGG without any visible fluorescence (107).

In a further step, Valdes et al. developed a novel fiberoptic probe connected to a spectrometer also for intraoperative use to measure intratumoral PpIX concentrations during surgery (101). The authors reported the first *in-vivo* application of this hand-held fiberoptic probe during surgery in a small patient cohort of 14 different brain tumors (101). In detail, the study cohort consisted of 2 LGG, 3 HGG, 6 meningiomas and 3 brain metastases (101). In this study, the PpIX concentration was measured intraoperatively during different time points of resection/intratumoral areas and corresponding tissue biopsies were collected (101). Additionally, control data were investigated from normal brain and dura mater (101). According to the data, the authors found significant differences in PpIX concentrations between all measured tumors and normal brain (101, 102). Therefore, this first *in-vivo* study demonstrated the feasibility of this innovative approach in different common brain tumors (101).

In a subsequent study published in 2015, Valdes et al. analyzed the value of their fiberoptic probe also in a small series of LGG including altogether 12 patients (34). In detail, two oligodendrogliomas, two gangliogliomas, one ependymoma, three dysembryoplastic neuroepithelial tumors (DNETs), three oligoastrocytomas, and one pleomorphic xanthoastrocytoma were included (34). According to the data, the authors confirmed the poor diagnostic accuracy of the conventional visual 5-ALA fluorescence technology in LGG (34). However, significant PpIX concentrations were measured in LGG by using the fiberoptic probe according to these preliminary data (34). In detail, quantitative fluorescence PpIX measurement with the intraoperative fiberoptic probe was able to markedly increase the diagnostic accuracy for detection of LGG tissue as compared to 38% for qualitative visible fluorescence (34).

Based on these promising preliminary data, Widhalm et al. applied this fiberoptic probe in addition to conventional visual 5-ALA fluorescence technology in a study published in 2019 during surgery of 22 suspected diffusely infiltrating LGG (35). In this study, final histology after surgical resection revealed a WHO grade II in 8 cases, WHO grade III glioma in 10 cases and WHO grade IV glioma in 3 cases (35). With assistance of 5-ALA visual 5-ALA microscopy, visible fluorescence was present in circumscribed areas in the majority of HGG (79%), whereas visible fluorescence was absent in all LGG (35). By using the fiberoptic probe, a significantly higher mean PpIX concentration was found in fluorescing samples as compared to non-fluorescing tissue samples (35). Furthermore, a significant correlation of PpIX concentrations and the percentage of tumor cells was observed in this study (35). Moreover, this study reported a significant correlation between the maximum PpIX concentration and overall tumor grade (35). Altogether, the authors conclude that conventional 5-ALA visual 5-ALA microscopy is especially useful to identify intratumoral anaplastic foci to avoid the risk of histopathological undergrading (35). The additional use of quantitative PpIX analysis represents a powerful technique for improved intraoperative detection of LGG tissue that is generally characterized by absence of visible fluorescence in order to maximize the extent of resection (35).

In a further study from 2019, Martinez-Moreno et al. applied a handheld spectroscopic probe in a series of 68 patients with diffusely infiltrating gliomas (WHO grades II-IV) (108). With *ex-vivo* spectroscopic analysis, significant differences in the median fluorescence intensity values were found in certain parameters of malignancy as well as a significant correlation between fluorescence intensity and proliferation rate (108). These data demonstrated the value of such spectroscopic probes to visualize intratumoral histopathological heterogeneity in diffusely infiltrating gliomas for improved tissue sampling and detection of the tumor margin (108).

## Confocal Microscopy and 5-ALA Fluorescence

Another innovative technique for improved detection of brain tumor tissue represents confocal microscopy combined with fluorescence technology (75, 109–113). In contrast to spectroscopic analysis, confocal microscopy is able to visualize fluorescing tissue by generating images with high contrast micron-scale spatial resolution (75, 109–113). The development of handheld probes provided new insight into the tumor enabling real time images of 5-ALA induced fluorescence at a 1000x magnification (75).

In 2011, Sanai et al. utilized confocal microscopy enabled through a handheld probe in order to visualize 5-ALA fluorescence during surgery of 10 LGG (WHO grades I and II gliomas) (75). Although none of the 10 LGG demonstrated visible fluorescence, confocal microscopy was able to visualize fluorescing tumor cells within LGG tissue (75). Therefore, the authors conclude that this innovative technique is useful to improve the detection of LGG tissue as well as their tumor margin in order to increase the rate of extent of resection (75).

Another approach was introduced by the group of Preul et al. with the combined use of intraoperative confocal laser endomicroscopy (CLE) and the fluorescence dye “fluorescein sodium” (110–113). In contrast to 5-ALA, this fluorescent dye is administered intravenously (110–113). By this innovative approach, the authors observed a high specificity and sensitivity for detection of glioma tissue (94% and 91%, respectively) and meningioma tissue (93% and 97%, respectively) comparable to those for conventional frozen sections (110–113). Therefore, the authors conclude that this technique will optimize in future surgery of brain tumors and intraoperative decision-making (110–113).

## Fluorescence Lifetime Imaging of PpIX

Apart from intensity-based and spectroscopic measurements fluorescence can be analyzed in respect to its temporal decay dynamics (114–117). The fluorescence lifetime is the average time delay between the excitation of a fluorophore and the subsequent emission of fluorescence (114–118). It depends both on the intrinsic molecular properties and the direct cellular environment (118). FLIM has been widely used to measure the cellular redox states of the coenzymes nicotinamide adenine dinucleotide (phosphate) (NAD(P)H) and flavin adenine dinucleotide (FAD), which have shown to indicate metabolic re-programming in cancer cells (119–121). FLIM for the sensitive detection of PpIX in photodynamic diagnosis (PDD), however, has gained interest only recently (114–117).

In intensity-based PDD the detection of weak PpIX fluorescence is limited by the autofluorescence of the brain parenchyma (122). When excited in the blue region of the spectrum, the main contributors to tissue autofluorescence emitting above 600nm are flavins, lipofuscin-like lipopigments, vitamin A and endogenous porphyrins (122). This background fluorescence can be stronger than the actual PpIX fluorescence (122). Sensitive detection therefore inherently requires the distinction between PpIX and autofluorescence, which essentially corresponds to the specificity of the PpIX detection (122). As described in the previous sections, spectroscopic and hyperspectral techniques have been proposed to quantify PpIX concentrations (35, 123, 124). FLIM of PpIX constitutes a complementary approach as it allows to distinguish spectrally overlapping fluorophores based on their temporal decay characteristics (35, 116, 123, 124). Autofluorescence and 5-ALA induced PpIX fluorescence lifetimes in an orthotopic mouse model have been reported by Kantelhardt et al. using a multiphoton time-domain FLIM system (125). While lifetimes from 0.8 to 2.0ns were measured for brain parenchyma, cytoplasmic PpIX increased the lifetime to 2.9 ns in U87 GBM derived cell lines (125). Autofluorescence in both murine white and gray matter was measured to be 1.4 ns, whereas cells with a higher metabolic activity exceeded those values (>1.7 ns) (126). Russel et al. measured the lifetime of PpIX to be 16.4ns in organic solvent and 6.3 ns for mitochondrial PpIX in rat prostate adenocarcinoma cells incubated with 5-ALA (127). Similarly, a lifetime of 7.4ns was reported in 5-ALA incubated epithelial rat cells (128). Erkkilae et al. recently proposed the use of a frequency-domain FLIM (FD-FLIM) system based on a dual-tap CMOS camera for *ex-vivo* imaging of human brain tumor specimens collected during 5-ALA fluorescence-guided resection (114). While a mean fluorescence lifetime of 15.1ns was measured for a GBM WHO grade IV, lifetimes of up to 4.8ns were reported for an oligodendroglioma WHO grade II in a follow up study (116). In an extended pilot study, the median of the measured lifetimes of 3 LGG was 2.5ns, where 100 randomly selected measurement points were evaluated for each specimen (115). Likewise, tumor infiltrated brain of LGG and HGG showed increased lifetimes (12 samples, 3.2ns). In contrast, a median autofluorescence lifetime of 1.9 ns was reported for 2 non-pathological specimens (115).

**Figure 2:**
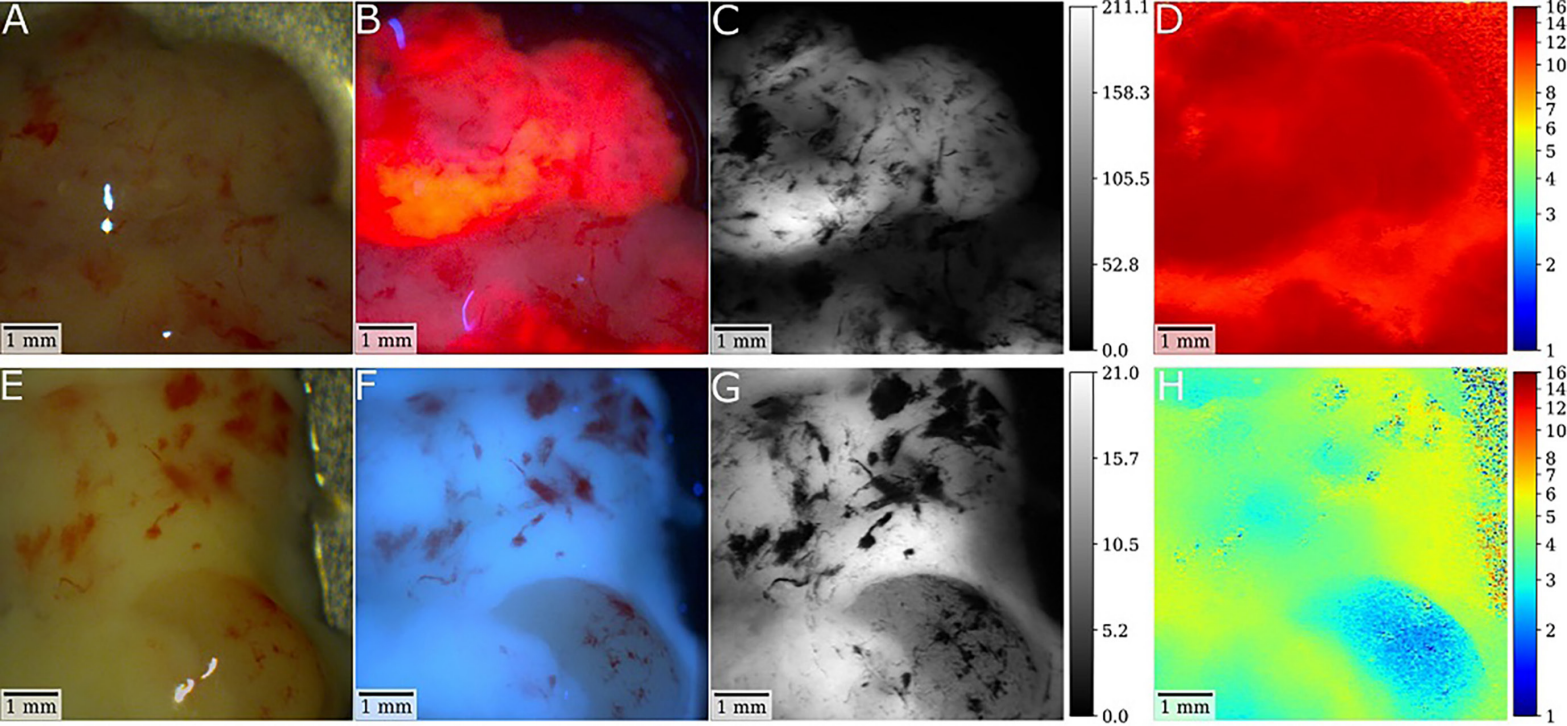
Illustrative cases of a glioblastoma (GBM) and low-grade glioma (LGG) with postsurgical *ex-vivo* analysis with fluorescence lifetime imaging after preoperative 5-ALA administration. **(A, B)** In the first patient, white-light and fluorescence camera images show the tumor core of an IDH-wild-type GBM specimen. For the fluorescence image, the laser was scanned rapidly across the tissue with the integration time of the camera being set to 2 seconds. Note the strong visible PpIX fluorescence of the sample. Demodulated fluorescence intensity [mV_RMS_] and fluorescence lifetime [ns] images of a raster-scanning FD-FLIM system are shown in **(C, D)**. As depicted by the color-coding, strong PpIX fluorescence led to increased lifetimes up to 15.3ns with a mean lifetime of 13.4ns. **(E, F)** In the second patient, respective images show a tissue specimen of an IDH mutated low-grade astrocytoma **(G, H)**. While no fluorescence was visible intraoperatively, the lifetime was increased from about 2.4 to 5.5ns, which is higher than the 0.8 to 2ns expected for non-pathological brain parenchyma. Thus, this tumor tissue could be visualized by fluorescence lifetime imaging.

FLIM of PpIX adds a third dimension to intensity-based and spectrally resolved techniques (117). Data suggest that weak PpIX accumulations, as found in LGG and infiltrated brain, increase the lifetime respective to non-pathological parenchyma. Further studies are required to determine the extent to which PpIX fluorescence can be detected within a dominating autofluorescence background. The homodyne detection scheme inherent to FD-FLIM facilitates the integration into surgical microscopes with long working distances (117). Clinical translation would therefore be consistent with surgical workflows and allow for combining sensitive wide-field PpIX detection with pre-operative stereotactic navigation data (117). Note that in FD-FLIM the measured lifetime is a weighted average composed of all excited fluorophores (117). Future studies should focus on combining spectrally-resolved methods with FD-FLIM to gain insight into the interplay of PpIX and autofluorescence. Also, time-domain FLIM techniques have been integrated into hand-held probes and could be used to reconstruct the multi-exponential decays of the mixed autofluorescence and PpIX signal (129, 130). Eventually, a combination of time- and spectrally resolved methods integrated into probes and wide-field visualization platforms could enhance LGG tissue detection in PDD and assist the neurosurgeon in a multitude of visualization tasks. An illustrative case is provided in **Figure 2**.

## Pharmaceuticals’ Influence on 5-ALA Fluorescence

The reason for the presence of visible fluorescence in pure LGG and in contrast, the absence of fluorescence in a group of gliomas with focal malignant histopathological characteristics are not fully clarified so far (25, 36, 58, 59, 131). The intake of antiepileptic drugs (AED) and/or dexamethasone prior to surgery has been discussed in the literature as a potentially influencing factor on 5-ALA fluorescence (94, 131–133). Two *in-vitro* studies analyzed the effect of different AED and/or dexamethasone on the metabolism of PpIX in malignant glioma cell lines (132, 133). The authors found a decreased amount of PpIX produced in these cells after the combination with dexamethasone as well as several AED, except Levetiracetam (132, 133). In line with these results, a first *in-vivo* study reported visible 5-ALA fluorescence more frequently in patients without preoperative intake of AED compared to patients with AED treatment in a series of 27 LGGs (94). However, this study did not only include diffusely infiltrating gliomas, but also ganglioglioma and pilocytic astrocytoma (94).

In a further study, Wadiura et al. investigated the influence of AED and dexamethasone on visible 5-ALA fluorescence in 110 newly diagnosed, suspected diffusely infiltrating LGG (131). According to the data, no independent correlation was found between the visible fluorescence status and the intake of dexamethasone/AED (131). According to these findings of the largest series to date, the treatment of AED/dexamethasone can be prescribed safely prior to fluorescence-guided surgery of LGG (131). However, future studies are warranted with the aim to detect even subtle alterations in intratumoral PpIX accumulation based on the potential influence of these frequently used drugs in neurosurgical patients using quantitative methods.

## Value of Other Fluorophores in LGG

Aside from 5-ALA other fluorophores were additionally investigated for fluorescence guided resection in HGG as well as LGG. One alternative to 5-ALA for intraoperative fluorescence represents sodium fluorescein (134–137). Sodium fluorescein is a technique for intraoperative fluorescence-guided resection in HGG, however, it showed no benefit in LGG surgery as no intraoperative yellow fluorescence was evident in LGG tissue (138, 139). A further fluorophore was investigated by Akimoto et al. analyzing talaporfin sodium for intraoperative photodiagnosis for malignant glioma (140). Aside from HGG and other tumor entities, also LGG cases were investigated in their study cohort and revealed at least weak fluorescence (140).

## Conclusion

The 5-ALA fluorescence technology is especially useful to visualize intratumoral regions with malignant transformation (anaplastic foci) within initially suspected LGG to avoid the risk of histopathological undergrading. However, the current 5-ALA technique is limited by the frequent absence of visible fluorescence within pure LGG. Recently, new approaches were introduced for improved detection of pure LGG tissue such as quantitative spectroscopic PpIX measurement, FLIM of PpIX and confocal microscopy. Consequently, further studies should clarify if these promising techniques are capable to reliably detect pure LGG tissue during surgery in order to maximize the extent of resection and improve the patient prognosis.

## Author Contributions

BK, JF, DR, LW, ME, AW, SH-J, MB, and GW substantially contributed to the conception and design of the work. BK, JF, DR, LW, and GW were responsible for data acquisition, analysis or interpretation of data for the work. BK, JF, DR, LW, ME, AW, SH-J, MB, and GW drafting the work or revising it critically for important intellectual content. All authors contributed to the article and approved the submitted version.

## Conflict of Interest

JF receives financial research support by NX Development Corp.

The remaining authors declare that the research was conducted in the absence of any commercial or financial relationships that could be construed as a potential conflict of interest.

## Publisher’s Note

All claims expressed in this article are solely those of the authors and do not necessarily represent those of their affiliated organizations, or those of the publisher, the editors and the reviewers. Any product that may be evaluated in this article, or claim that may be made by its manufacturer, is not guaranteed or endorsed by the publisher.

## Abbreviations

5-ALA, 5-aminolevulinic acid; AED, Antiepileptic drugs; ATRX, Alpha thalassemia/intellectual disability X-linked; DNET, Dysembryoplastic neuroepithelial tumor; CLE, Confocal laser endomicroscopy; CMOS, Complementary metal-oxide semiconductor; EOR, Extent of resection; FAD, Flavin adenine dinucleotide; FDA, U.S. Food and Drug administration; FD-FLIM, Frequency-domain-FLIM; FET, ^18^F-fluoroethyl-L-tyrosine; FLAIR, Fluid-attenuated inversion recovery; FLIM, Fluorescence lifetime imaging; GBM, Glioblastoma; HGG, High-grade glioma; IDH, Isocitrate dehydrogenase; LGG, Low-grade glioma; MET, ^11^C‐methionine; MGMT, O6-methylguanine-methyltransferase; MRI, Magnetic resonance imaging; NAD(P)H, Nicotinamide adenine dinucleotide (phosphate); PDD, Photodynamic diagnosis; PET, Positron emission tomography; PpIX, Protoporphyrin IX.
